# Cochlear Implant Induced Labyrinthine Ossificans in Mondini Malformation: A Case Series

**DOI:** 10.7759/cureus.32648

**Published:** 2022-12-17

**Authors:** Abdulaziz Alsalhi, Yazeed A Alshawi, Hamad S Alsalhi, Abdulrahman Hagr

**Affiliations:** 1 Dermatology, King Saud University, Riyadh, SAU; 2 Otorhinolaryngology - Head and Neck, Prince Sultan Military Medical Hospital, Riyadh, SAU; 3 Family and Community Medicine, Security Forces Hospital, Riyadh, SAU; 4 Otorhinolaryngology - Head and Neck, King Abdullah Ear Specialist Centre (KAESC) King Abdulaziz University Hospital, King Saud University, Riyadh, SAU

**Keywords:** sensorineural hearing loss, hearing implants, ent procedures, bilateral hearing loss, implantation otology

## Abstract

Cochlear implantation is relatively a safe procedure with a favorable outcome. Labyrinthine ossification is one of the rare complications that has been observed in some occasions post-cochlear implantation. This paper report two cases of Mondini inner ear malformation cochlear implant failure associated with labyrinthine ossification, mandating revision surgery, and a literature review focusing on the reported cases, risk factors, surgical and non-surgical measures to mitigate this complication and to improve overall cochlear implant outcomes.

## Introduction

Hearing loss is associated with a major social burden, and it has implications for psychosocial health, quality of life, and economic independence [1]. Recent estimates from the World Health Organization (WHO) indicate that 466 million people suffer from hearing loss, and this figure is projected to rise to 630 million and 900 million by 2030 and 2050, respectively [2]. At present, 34 million children suffer from hearing loss, and newborn screening programs report a deafness rate of 1.33 per 1,000 [3]. Cochlear implant (CI) has profoundly affected the history of hearing loss since the first successful procedure was performed in 1961 by William House and John Doyle [4]. These surgically implanted devices can solve severe-to-profound sensorineural hearing loss, provided that the retrocochlear pathway is intact.

Labyrinthine ossificans (LO) is considered an acquired factor that, in certain cases, can be anticipated and avoided, in which fibrosis and neo-ossifications replace the perilymph due to infectious or traumatic processes [5]. Mondini malformation (commonly known as Mondini dysplasia), which is classified by Sennaroglu and Saatci as an incomplete partition type 2 (IP-II), is the most common type of inner ear malformation (IEM), which occurs in around 7.5-8.8% of pediatric patients with CI [6-8]. In 1791, Mondini reported the first case of IP-II, which was described as being characterized by a cochlea shortened to only one and a half turns, a dilated vestibule, and an enlarged vestibular aqueduct. The first CI used to treat IEM was in 1983 with the Mondini dysplastic ear, and since then, the presence of IP-II is no longer an obstacle for surgical interventions of this kind [9]. Interestingly, the long-term outcomes of CI treatment for Mondini dysplasia are favorable, and patients experience a similar level of hearing compared to their matched peers with no IEM [10,11].

This case series presents the cases of two patients with complete cochlear ossification following CI in ears with Mondini malformation. In the first case, a rare association is observed involving bilateral Mondini malformation, as well as right normal and left severe internal auditory canal (IAC) hypoplasia with no identifiable nerves passing through. The second case involves bilateral Mondini malformation with LO in the left ear, which happened to be more severely malformed.

## Case presentation

First case

A full-term four-year-old boy, who is not known to have any medical illness apart from left congenital facial paralysis since birth, presented at our otology clinic with a complaint of delayed language development. The patient’s family noticed that he was not responsive to sound. With the exception of an episode of acute *Haemophilus influenzae* meningitis at the age of 2, for which the patient had been hospitalized for 10 days and treated with intravenous antibiotics, the medical history was unremarkable. The patient presented with no other otological, vestibular, or nasal complaints. 

Physical examination revealed a healthy-looking child with left grade 5 House-Brackmann facial weaknesses. Evaluation of bilateral pinnae, external auditory canals, and tympanic membranes were all within normal limits, as well as nose, throat, and cranial nerves, with the exception of left lower motor neuron facial nerve weakness as mentioned above. Audiological investigation revealed the absence of bilateral distortion product otoacoustic emissions. Auditory brain stem response showed no wave 5 detected down to 90 dBnHL bilaterally. 

**Figure 1:**
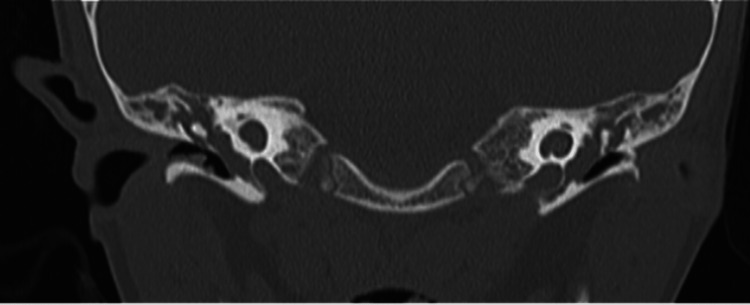
CT temporal bone coronal view showing bilateral cystic vestibule and bilateral cystic apical and middle turns of the cochlea.

**Figure 2:**
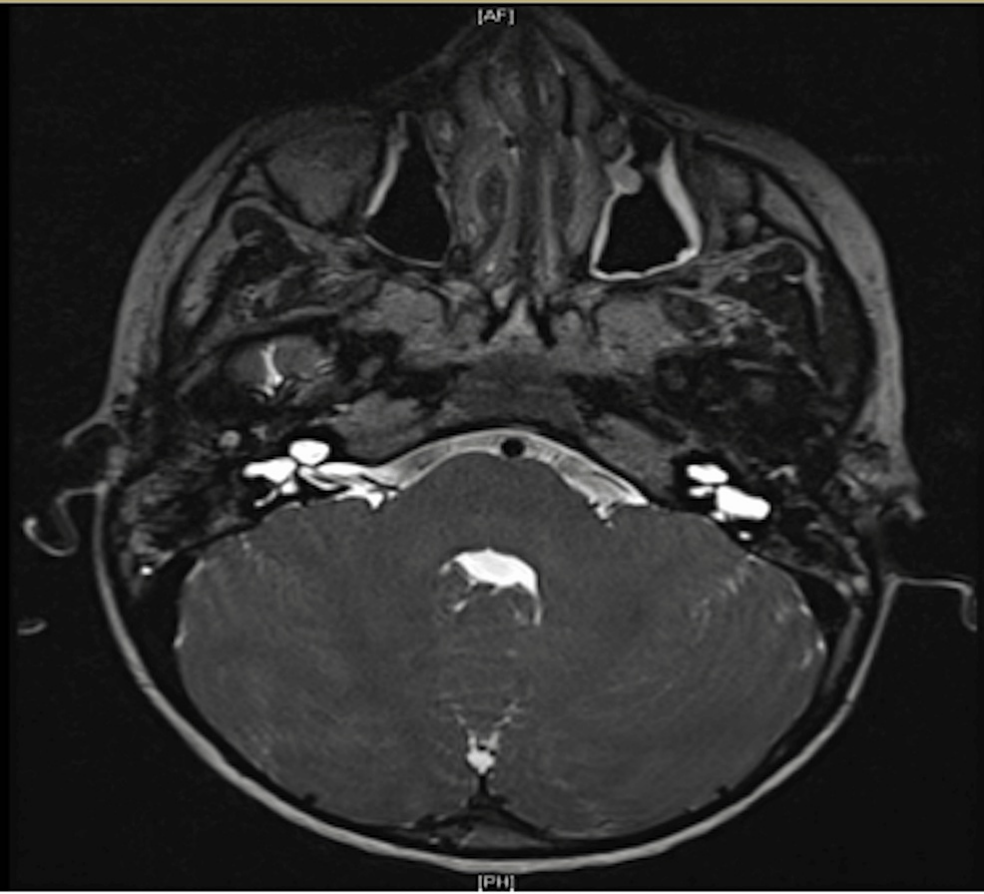
MRI T2-weighted image at the level of internal auditory canal showing atresia of the left internal auditory canal with no identifiable cochlear nerve. Right cochlear nerve is identified passing through the right auditory canal. Bilateral cystic vestibule and bilateral cystic apical and middle turns of the cochlea.

Non-contrast temporal computed tomography (CT) revealed a hypoplastic IAC on the left side, normal-looking right IAC with bilateral cochlear incomplete partition type II (IP-II), large vestibular aqueduct, and cystic vestibule (Figure 1). Magnetic resonance imaging (MRI) of the IAC revealed an absence of the seventh and eighth cranial nerves on the left side, while these cranial nerves were clearly identified in the right side. However, the cochlear nerve on the right side was hypoplastic (Figure 2). MRI image analysis indicated that both cochleae were consistent with bilateral Mondini malformation, and the T2 fluid signal were normal.

The patient underwent right-sided CI under general anesthesia. Using post-auricular skin incision, an anteriorly based palva flap was created, followed by cortical mastoidectomy and device bed creation. Through posterior tympanotomy (facial recess), the round window was partially opened and full smooth insertion of MED-EL FLEX 28 electrode (MED-EL, Innsbruck, Austria) was performed. Mild perilymph oozing was observed after opening the round window, which was easily managed by head elevation. The round window opening was sealed around the electrode with fascia. Intraoperative impedances and electrically evoked compound action potential (eCAP) were all within normal ranges. The patient tolerated the procedure well, and the postoperative course was uneventful. On the first postoperative day, the patient was vitally stable and playful, and after a wound examination with unremarkable findings, he was discharged home. Post-operative X-ray confirmed the full insertion of the intracochlear electrode. 

At the beginning of the second postoperative week, the CI was switched on. Afterward, the patient received regular follow-up appointments with our hospital’s audiology, speech, and ENT departments for one year, and he showed acceptable improvement in speech and academic attainment. Following this, he was lost to follow-up for six years, after which he presented with a progressive deterioration of CI function. There was no history of ear infections or meningitis. Impedance field telemetry (IFT) showed high impedance at electrodes 5, 6, and 7, which indicated that these were deactivated, and therefore new maps were provided. Despite normal full ENT examination, the patient’s auditory function did not improve. IFT was repeated and nine open circuits were identified with no eCAP, both of which are indicative of device failure. 

**Figure 3:**
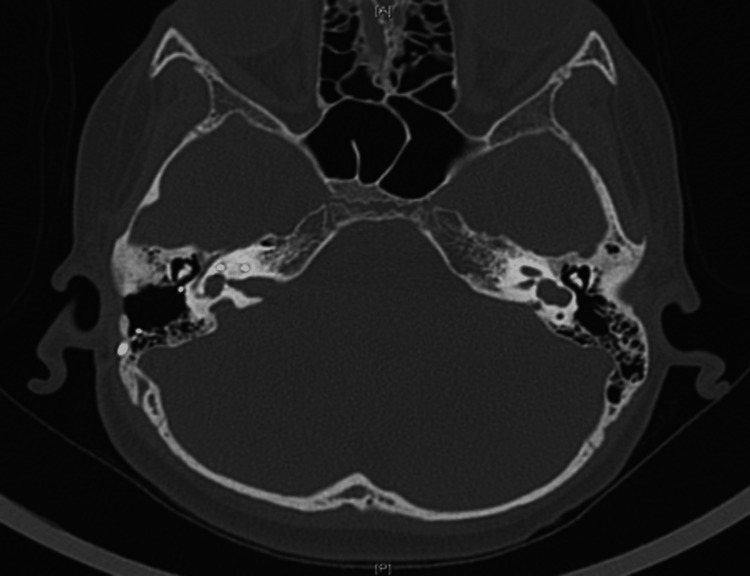
CT temporal bone axial cut showing near-complete right labyrinthine ossification around the electrode; CI electrode is intact within the cochlea. On the left, there is severe stenosis of the lateral and atretic medial part of left internal auditory canal. CI, cochlear implant

**Figure 4:**
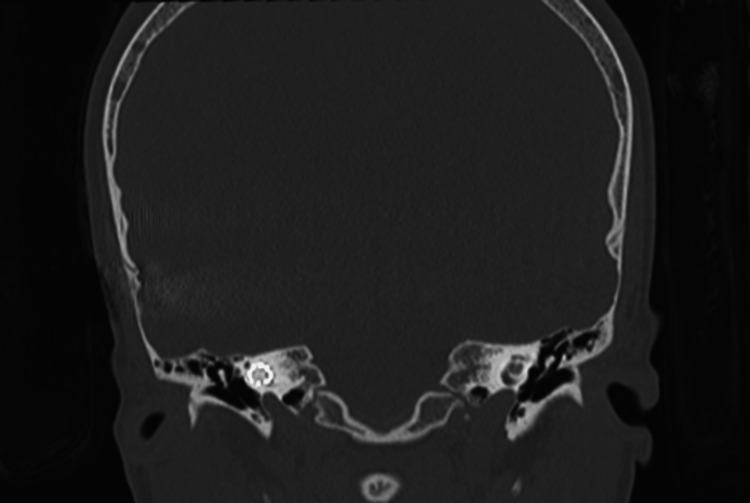
CT temporal bone coronal view of the same patient showing right cochlear labyrinthine ossificans.

CT imaging revealed partial right cochlear ossification, primarily beyond the first turn of the cochlea (which seemed to compress the distal electrodes), which was suggestive of LO (Figures 3, 4). However, no changes were observed in the left cochlea, including no ossifications, no fracture lines, and no destructive lesions in the Petrus or the skull base. After discussing the case with the Cochlear Implant Committee, the decision to undertake CI revision surgery was made. This decision was principally based on the aplasia of the left cochlear nerve. Unfortunately, intraoperative assessment of the middle ear through posterior tympanotomy indicated that the electrodes were cut due to the ossifications, and only the lateral half was extricated from the round window. Both attempts to insert through the round window, as well as to drill out the cochlea, were unsuccessful. At this point, the surgery was aborted. The patient tolerated surgery well, and upon discharge on the second postoperative day, the family was counselled for auditory brainstem implantation. At present, the patient is on the waiting list for this intervention.

Second case

A full-term 15-month-old boy presented to our clinic with a complaint of delayed language development. The patient failed the neonatal hearing test, and had a history of repaired cleft lip and palate, Duane syndrome, and attention deficit hyperactivity disorder (ADHD). Physical examination revealed a healthy-looking child with a normal head and neck examination, apart from a scar from the cleft lip and palate repair. Audiological investigation revealed the absence of bilateral distortion product otoacoustic emissions. Auditory brain stem response showed no wave 5 detected up to 90 dB bilaterally. The patient failed to show any improvement with powerful hearing aids. 

**Figure 5:**
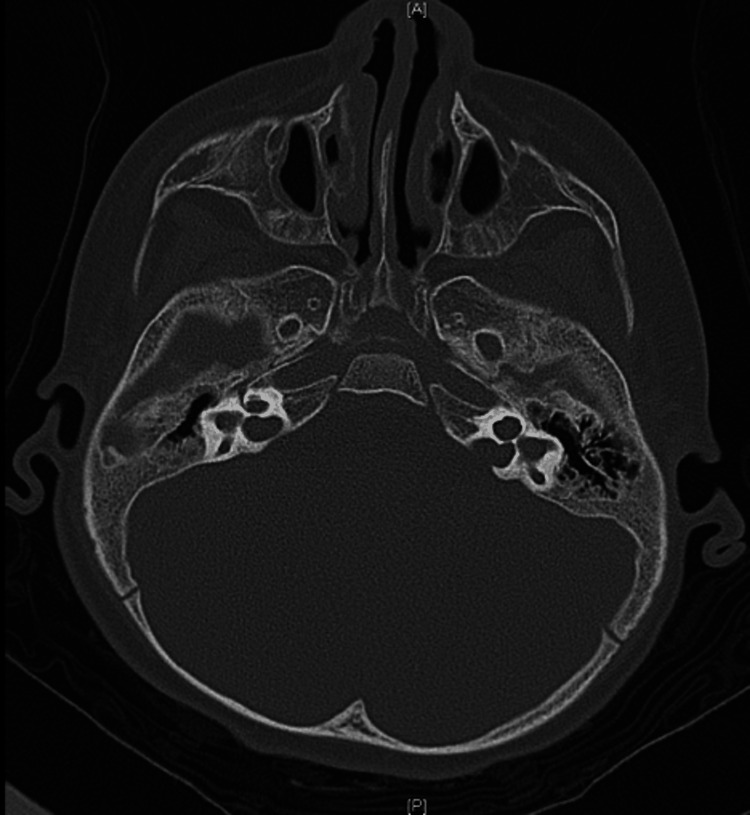
CT temporal bone axial cut showing patent bilateral internal auditory canals with dilated cystic vestibules, and dilated cochlear middle and apical cochlear turns.

Non-contrasted temporal CT revealed a normal-looking bilateral IAC with bilateral Mondini malformation, which seemed to be more severe on the left side (Figure 5). MRI of the IAC revealed healthy-looking seventh and eighth cranial nerves. Mild cystic dilatation of cochlea, vestibule, and semi-circular canals bilaterally were also observed. Furthermore, the left cochlea had less than one turn, while the right cochlea had one and a half turns. MRI images were consistent with bilateral Mondini malformation, and the T2 fluid signal was normal.

After counseling the patient’s family and receiving approval from the Cochlear Implant Committee, the patient received general anesthesia and underwent bilateral CI at age of three years. The same surgical techniques reported in the first case were applied, with the exception that the deep round window for this patient precluded visualization, and thus cochleostomy was undertaken. Insertion of MED-EL FORM 24 electrodes was confirmed intraoperatively using Mastoid X-ray. Identical steps were followed for the left ear, where a cochleostomy was again performed due to the inability to visualize the round window. The patient tolerated surgery well and was doing well with no complaints. 

The CI was switched on few days later. The patient displayed positive improvements in language until 10 months, at which point the level of improvement plateaued. Auditory response telemetry was undertaken, revealing that the right ear was responsive, while the left ear, with the exception of the first and second channels, was unresponsive. Professional IFT was conducted and was reassuring bilaterally. Accordingly, mapping and counseling with the parents were undertaken. One month later, the patient became uncooperative during speech sessions, unable to stay still and focus. He was diagnosed with ADHD, for which risperidone was prescribed. Following this, patient performance and compliance with speech sessions improved. Six months later, a reduction in the rate of speech improvement was noticed, and thus IFT was repeated, which revealed normal right but moderately elevated impedances in the left ear. New maps were given in combination with close follow-ups. However, two months later, left implant electrodes 4 and 12, as well as ground path impedance, increased, and device failure was suspected. 

**Figure 6:**
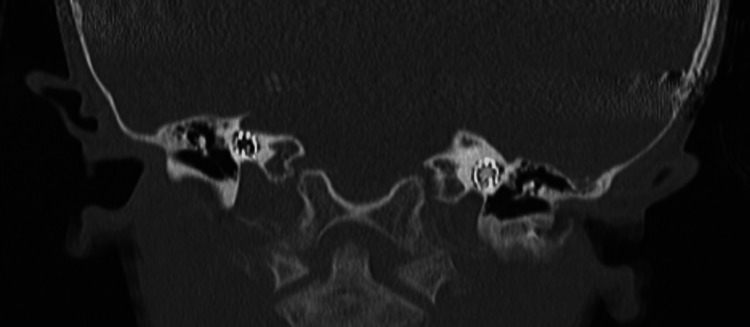
CT temporal bone coronal view showing left cochlear near complete ossification; the right cochlea is healthy-looking. Both cochlear implant electrodes are intact within the cochlea.

The patient underwent temporal CT, which revealed left cochlear and semi-circular canal ossifications. This was considered indicative of LO. However, the right cochlea was healthy-looking, and both devices were in place (Figure 6). IFT was repeated, indicating high impedance in electrodes 4, 7, 9, and 12, for which it was deactivated. Additionally, ground path impedance remained high. IFT readings for the right ear were normal. The patient is currently scheduled for left revision CI.

## Discussion

The two cases presented above show CI failure due to LO. Every effort to restore the patient’s hearing was unsuccessful in the first case. LO is a pathological process that leads to the complete ossification and obliteration of the cochlea and vestibular system. The pathogenesis of the condition is separated into three phases: firstly, acute inflammation; secondly, fibrosis; and, finally, new formation of pathological ossification of the membranous labyrinth [11]. In some animals, the time elapsed between the insult and the first evidence of ossification can be as short as two months, but this period seems to be longer in humans [12]. Several etiologies can lead to LO, including infection, cochlear fracture, intralabyrinthine hemorrhage, autoimmune diseases of the inner ear, and tumors. Infection is the most common identifiable cause, and suppurative labyrinthitis is the result of microbial invasion of the inner ear through three major pathways: meningeal, hematological, and tympanic extension [13]. 

The first patient endured an acute episode of *H. influenza* meningitis at the age of 2 years, and the question of whether this contributed to their loss of hearing is difficult to judge. However, the patient’s linguistic competence did not develop prior to the bout of meningitis, and his family denied that he was ever responsive to sounds. Both considerations support arguments against post-meningitis hearing loss. Moreover, it is known that patients with Mondini malformation are at a higher risk of recurrent meningitis and its sequelae. Nevertheless, the fact remains that this patient only experienced one episode of meningitis more than 9 years previously. There was no significant occurrence of gusher in the first surgery and no signs of gusher in the revision, both of which suggest that meningitis is not a likely cause [14]. Moreover, unilateral LO does not support meningitis as a cause. In fact, it points toward other ear-specific etiologies. 

Speech improvement for the second patient fluctuated. In accounting for this result, it is relevant to highlight the possibility that this patient’s ADHD interfered with accurate assessment. Despite having received bilateral CI, the patient’s left ear showed audiological and radiological evidence of implant failure. There were no identifiable causes of this unilaterality, and the patient’s history and medical records did not suggest potential causes. The possibility that the left cochlea’s more severe malformation was the cause of LO on this side remains a topic of debate. 

A well-known association exists between the surgical implantation of CI devices and LO. As observed in both human and animal trials, a key factor implicated in this association is the trauma arising from electrode insertion (e.g., dissection of the spiral ligament, fracture or dislocation of the osseous spiral lamina, and displacement or perforation of the basilar membrane). However, delayed inflammatory responses may play a role because a CI device is ultimately a foreign body [15,16]. Although the observed new formation of bone is insufficient evidence to prove causation, several studies indicate that CI ears are more likely to develop ossification compared to non-CI ears [14]. Moreover, ossification typically surrounds the site of maximal trauma during electrode insertion. Histopathological studies have offered evidence of new bone formation over electrodes [15]. Additionally, post-CI LO affects hearing quality and word recognition, and histopathological studies have shown a reduction of spiral ganglion cells in these ears [15,17].

Kamakura and Nadol demonstrated that all examined CI patients (n = 17) showed some degree of ossification and fibrosis, and consonant-nucleus vowel-consonant (CNC) word scores were negatively correlated with ossification but not fibrosis [16]. Gutiérrez-Salazar et al. reported that 3.6% of the decisions made to re-implant a CI device were informed by LO [18]. Richard et al. compared different surgical approaches and found that the round window insertion technique was associated with the lowest incidence of cochlear ossification [19]. It worth mentioning that the round window insertion technique was performed in the first case, while cochleostomy was undertaken in the second case. A recent animal clinical trial showed a significantly lower level of fibrosis and ossification, paired with improved hearing, after cochleostomy in the group that received intracochlear dexamethasone compared to a systemic and no dexamethasone group [20]. 

We reported these two cases to shed light on manifestations of this uncommon complication of CI. We hope to raise the awareness of the audiological presentation of LO and to stress the importance of a low threshold for CT temporal bone in patients with suspected CI device failure. We recommend both surgical and non-surgical measures to mitigate these complications and to improve overall CI outcomes. Atraumatic soft electrode insertion techniques are worth pursuing, as well as the sealing of surgical entry site with fascia or electrode stopper to prevent perilymphatic oozing, especially in malformed ears. Maintaining a sterile surgical field and applying homeostatic measures are also important. Surgical entry through the round window is thought to result in less structural damage and fibrosis. The importance of vaccines and early recognition of ear and meningeal infections is also worth emphasizing. Further development of electrodes that are flexible and biocompatible, and which also have a patient-specific cochlear duct length, is critical. This is particularly true when Mondini malformation is identified, since the cochlear duct is typically shorter. This means that part of the electrode is coiled at the duct apex. Finally, it is necessary to train audiologists to handle difficult patients, where the recognition of a decrease in hearing is often challenging. Since there are no reported measures to decrease such complications after CI in malformed inner ears in the literature, we recommend further research and investigation.

## Conclusions

LO is a serious complication that may arise following CI. Many potential causes must be considered when encountering LO in clinical settings, and it is essential to understand the available preventive measures and clinical pathways for treatment and management. There should be a low index of suspicion for such a complication, even in presence of other potential causes of slow speech improvement (e.g., ADHD). Many factors contribute to the development of CI-induced LO, including traumatic electrode insertion, cochleostomy, post-implantation meningitis, and autoimmune diseases (e.g., otosclerosis). Although ossification and fibrosis are not sources of hazard in some cases, they can lead to device failure and irreversible cochlea damage. Further studies should investigate the impact of CI on LO in both normal and malformed inner ears.
